# Multifunctional Evaluation Technology for Diagnosing Malfunctions of Regional Pelvic Floor Muscles Based on Stretchable Electrode Array Probe

**DOI:** 10.3390/diagnostics13061158

**Published:** 2023-03-17

**Authors:** Shengming Wang, Luoqi Yang, Haofei Jiang, Jie Xia, Wenjuan Li, Zujuan Zhang, Shaomin Zhang, Hao Jin, Jikui Luo, Shurong Dong, Yanlan Yu, Zhenwei Xie

**Affiliations:** 1 Key Lab. of Advanced Micro/Nano Electronic Devices & Smart Systems of Zhejiang, College of Information Science and Electronic Engineering, Zhejiang University, Hangzhou 310027, China; 2 Department of Gynecology, Women’s Hospital, Zhejiang University School of Medicine, Hangzhou 310006, China; 3 Department of Urology, Sir Run Run Shaw Hospital, School of Medicine, Zhejiang University, Hangzhou 310016, China; 4 Key Lab. of Biomedical Engineering of Ministry of Education, Qiushi Academy for Advanced Studies, Zhejiang University, Hangzhou 310027, China

**Keywords:** point-of-care diagnostics, multifunctional evaluation technology, stretchable electrode array probe, regional dysfunction diagnosis, inter-region correlation, pelvic floor muscle

## Abstract

The pelvic floor dysfunction (PFD) has become a serious public health problem. Accurate diagnosis of regional pelvic floor muscle (PFM) malfunctions is vitally important for the prevention and treatment of PFD. However, there is a lack of reliable diagnostic devices to evaluate and diagnose regional PFM abnormality. In this work, we developed a multifunctional evaluation technology (MET) based on a novel airbag-type stretchable electrode array probe (ASEA) for the diagnosis of malfunctions of regional PFM. The inflatable ASEA has specifically distributed 32 electrodes along the muscles, and is able to adapt to different human bodies for tight contact with the muscles. These allow synchronous collection of high-quality multi-channel surface electromyography (MC-sEMG) signals, and then are used to diagnose regional PFM malfunctions and evaluate inter-regional correlation. Clinical trial was conducted on 15 postpartum stress urinary incontinence (PSUI) patients and 15 matched asymptomatic women. Results showed that SUI patients responded slowly to the command and have symptoms of muscle strength degeneration. The results were consistent with the relevant clinical manifestations, and proved the reliability of MET for multifunctional PFM evaluation. Furthermore, the MET can diagnose malfunctions of regional PFM, which is inaccessible with existing technology. The results also showed that the dysfunction of PSUI patients is mainly located in iliococcygeus, pubococcygeus, and urethral sphincter regions, and there is a weak correlation between these specific regions and nearby regions. In conclusion, MET provides a point-of-care diagnostic method for abnormal function of regional PFM, which has a potential for the targeted point-to-point electrical stimulation treatment and PFD pathology research.

## 1. Introduction

Pelvic floor dysfunction (PFD) is a common disease with high incidence among women and has become one of the serious public health problems [1,2]. Damages in the female pelvic floor muscles (PFM) is one of the main causes of PFD [3,4]. PFM has complex structures [2], and PFM-associated diseases often involve different functional defects of regional muscle groups. For example, the deficiency of urinary sphincter and weak urethra-supported strength may cause the stress urinary incontinence (SUI) [5]; anal sphincter injury are the most common cause of anal incontinence in women [6]; the avulsion of obstetrical levator ani muscle and larger levator hiatus are associated with pelvic organ prolapse [7]. Therefore, it is necessary to conduct detailed research on abnormal regions and functions of PFD-related muscles, so that intervention strategies can be established for treatment of specific PFD diseases.

At present, specific functional information of PFM can be obtained using various detection systems. Ultrasound is a well-used tool to check the disease status [8,9,10]. Magnetic resonance imaging technology can also be applied to detect the symmetry and coordination of PFM [11]. These clinical systems are large and expensive, and require skilled personnel to operate, which cannot work efficiently under the limited condition of space and personnel resources, resulting in a long time test period. At the same time, each test costs patient almost hundreds of money. There are also some convenient devices for real-time evaluation and diagnosis. For example, the vaginal pressure airbag is a common device for evaluating PFM force [12,13]. Handheld devices are used for measuring the mechanical properties of PFM [14]. The surface electromyogram (sEMG) is the main technology for evaluating the PFM activity status in clinic [8,15,16,17]. However, traditional rigid dual-channel probe is used to collect the sEMG signal of PFM, which cannot characterize the complex PFM structure because it can only collect the sEMG signal of integral PFM rather than the detailed information of the muscles [18,19]. Some researchers use the rigid high-density probe array with equal distance distribution to collect more information [20,21]. Since the probes were made from rigid materials and had fixed shapes and contact pad locations, the contact pads of the probes could not make tight contact with mucosa for the necessary assessment due to irregular shape of the vagina, which are very likely to shift during tests, and even to be pushed out of the vagina during contraction assessment. Overall, there is a lack of reliable point-of-care diagnostic device to obtain comprehensive PFM function and conduct in-depth regional diagnosis.

Recently, we developed a multifunctional evaluation technology (MET) based on an airbag-type stretchable probe (ASEA) [22] to diagnose the malfunctions of regional PFM. The ASEA is able to be inflated, so that it can fit vaginal cavities of different sizes and shapes. It has multiple electrode arrays specifically arranged according to the muscle distribution. These allow the multiple electrodes to make tight contact with muscles for accurate MC-sEMG signal acquisition, and subsequent comprehensive assessment and diagnosis of functions of regional PFM [23,24], provided a new method and strategy for diagnosis and treatment of PFD diseases.

In this paper, we report on MET-based assessment of SUI. SUI is one of the common PFD, and delivery is one of the risk factors for its occurrence. Therefore, postpartum stress urinary incontinence (PSUI) in patients is an important group of SUI prevention and treatment. Fifteen PSUI patients and fifteen postpartum asymptomatic women with matching personal information participated in the clinical trial. They conducted Glazer protocol [25,26] and we applied MET to comparing the evaluation parameters. Results showed that the PSUI patients had weaker PFM reactivity and strength than those of the health control group, and the type-I and type-II muscle dysfunctional regions are mostly located in the iliococcygeus, pubococcygeus, and urethral sphincter regions. There is a weak correlation between these regions and the nearby muscle groups. These results are consistent with the SUI pathogenesis theory. The MET is able to obtain detailed information of regional PFM that the existing devices are unable to provide. The work demonstrated the excellent diagnosis ability of the multifunctional evaluation technology, and application potential for personalized treatment and rehabilitation.

## 2. Materials and Methods

### 2.1. Participants

A clinical trial was conducted to investigate the PFM regional function under different states. The trial was conducted from January to September 2021. Female patients were recruited from the department of Gynecology and Obstetrics, Women’s hospital school of medicine Zhejiang University and asymptomatic volunteers were responded to a notice published in the poster. A total of 30 postpartum women were recruited for the evaluation. All participants were of childbearing age and older than 18 years, were sexually active, and can tolerate vaginal examinations. Participants with the following conditions were excluded: pregnant; lithiasis of urinary system; acute inflammation of pelvic organs and vagina; lower urinary tract obstruction and kidney disease; vaginal bleeding; history of pelvic malignant tumor and pelvic radiotherapy; nervous system diseases that obviously affect muscle function; history of operation about urinary incontinence; pelvic organ prolapse.

They were divided into two groups: postpartum and asymptomatic, control group (Group A) and PSUI group (Group B). The group A women had given birth within one year without PFD-related symptoms disease; but the group B women had the symptom of involuntary leakage of urine when coughing, sneezing etc. Demographic information showed no statistical difference between the two groups as summarized in Table 1.

The clinical trials in this work have been approved by the Ethics Committee of the Women’s Hospital, School of Medicine, Zhejiang University, Hangzhou, China, ID: No. 067 (2019), and the study was conducted in accordance with the ethical principles of the Declaration of Helsinki. All participants provided written and informed consent. Each participant had been informed about the protocol and signed the informed consent before the trial. All the participants were trained to perform the proper contraction of the PFM.

### 2.2. Airbag-Type Stretchable Electrode Array Probe

**Figure 1:**
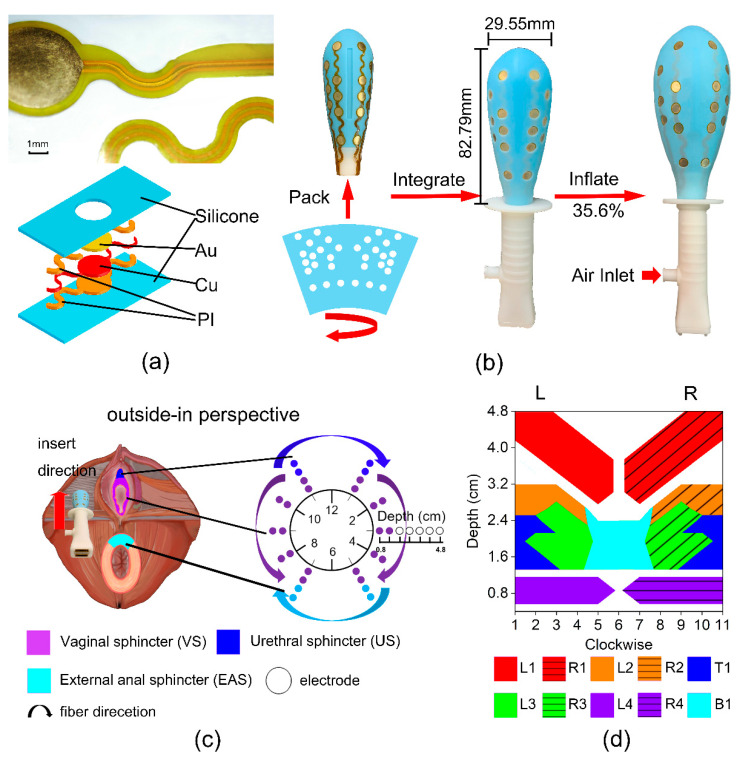
The ASEA and 10 PFM regions and distribution. (**a**) The localized flexible circuit image under a microscope (upper) and explosive structure (bottom) of ASEA. (**b**) The macro internal structure of ASEA (left), the ASEA before inflation (middle) and after inflation (right). (**c**) The concept map of ASEA collecting sEMG signal. The left figure is part of the PFM distribution from the outside-in perspective. ASEA is inserted from the outside of the screen to the inside. The right figure shows the clockwise, and the corresponding electrode layout direction. (**d**) The distribution map of 10 PFM regions based on physiology and PFM anatomy. Different regions are distinguished by different colors.

The ASEA is an airbag-type inflatable probe. The structure of the electrode of ASEA is shown in Figure 1a, and the configuration of the ASEA is shown in Figure 1b. The ASEA consists of a large number of bipolar electrodes placed along the muscle fibers, enabling the accurate signal acquisition from specific muscles. Air was injected into the air inlet (at the lower left corner) to expand the ASEA, and the pressure value in the airbag can be recorded, so the ASEA can also be used for evaluating the muscle-pressure feedback during PFM contraction. ASEA can expand up to 35.6% in volume, thus it is able to fit different sizes of the vagina. This configuration ensures a stable probe-muscle contact interface, and is able to follow the movement of muscle during muscle contraction, so that the ASEA can collect high-quality sEMG signals [27,28]. The fabrication process and details of the ASEA device are presented in our previous paper, and the results have demonstrated that the accuracy and stability of ASEA device are far superior to existing PFM electrode probes [22]. The design comparison between ASEA-based sEMG acquisition method and existing technologies can be found in Note S1, Figure S1 and Table S1 (Supplementary Materials). The price of disposable ASEA was about 40 yuan, even lower than the selling price of commercial rigid probe. The cost comparison between ASEA-based sEMG acquisition method and existing technologies can be found in Table S2. For convenience, the device structure, electrode arrangement, and structures are all shown in Figure.

The arrangement of the electrodes are based on the muscle distribution obtained by the physiology and PFM anatomy. With the assistance of tomography ultrasound imaging technology, we acquired images of anatomical structures of pelvic floor in different sections and investigated the muscle distribution. Based on these, it is possible to know clearly the direction of the muscle fiber and the range. According to the approximate muscle distribution and its fiber direction, we placed a large number of electrodes along with the PFM fiber directions for the collection of high quality sEMG. As shown in Figure 1c, the circles represent the electrodes and the arrows represent the muscle fiber direction. Twenty-four electrode pairs form a ten regions distribution map, including L1 (1-5 o’clockwise, 3.2–4 cm depth), L2 (1-4 o’clockwise, 2.8–3.2 cm depth), L3 (2-4 o’clockwise, 1.6–2.8 cm depth), L4 (1-5 o’clockwise, 0.8 cm depth), R1 (7-11 o’clockwise, 3.2–4 cm depth), R2 (8-11 o’clockwise, 2.8–3.2 cm depth), R3 (8-10 o’clockwise, 1.6–2.8 cm depth), R4 (7-11 o’clockwise, 0.8 cm depth), T1 (9-12,0-3 o’clockwise, 1.6–2.4 cm depth), B1 (5-7 o’clockwise, 1.6–2.4 cm depth), as shown in Figure 1d. According to the ultrasonic-assisted measurement, the muscle relationship corresponding to these regions are: T1: urethral sphincter (US); B1: external anal sphincter (EAS); L1, R1: iliococcygeus muscle (IC); L2, R2: pubococcygeus muscle (PC); L3, R3: puborectalis muscle (PR); L4, R4: vaginal sphincter (VS).

### 2.3. Evaluation System in MET

**Figure 2:**
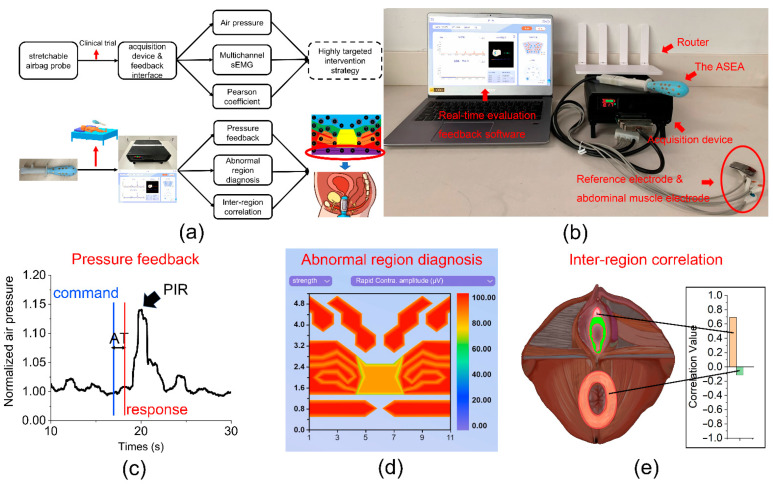
Detailed information of the MET. (**a**) The evaluation system framework of MET. The pressure and MC-sEMG data collected by ASEA based on Glazer protocol are submitted to the acquisition device and feedback interface for processing, and obtained the results of pressure feedback, abnormal area diagnosis contour map and interregional correlation. Targeted treatment strategy can be implemented in malfunction region. (**b**) System physical diagram. (**c**) Air pressure feedback waveform. AT is the time difference between the start of command (blue) and the start of response action (red). PIR is the growth rate of the air pressure in the contraction phase compared with the initial static air pressure. (**d**) PFM abnormal region diagnosis module. The functional parameter values of each region were presented on the contour map, and the color differences make the abnormal regions easy to be identified. (**e**) Inter-regional correlation analysis module. After determining the abnormal region, calculate the correlation value between the area and the nearby regions. The larger absolute value represented the closer relationship, and the positive and negative signs represented the cooperation and confrontation relationship.

The evaluation system framework of MET is shown in Figure 2a. The air pressure signal and MC-sEMG signal collected by ASEA are transmitted to the acquisition device. The pressure signal can be analyzed directly, and the sEMG signal is divided into short sections with a 1000 ms length, and is processed by a root mean square (RMS). A 20–400 Hz bandpass filter is applied to remove all power frequency interference. All data are processed by Matlab (Version 2021b). Then, the interface analyzes the pressure feedback data to evaluate the mechanical properties of PFM, evaluates muscle function and diagnoses abnormal PFM region according to the relevant sEMG parameters, identify the specific region and calculates the inter-region correlation. Finally, we analyze the cause of abnormal state at the level of synergy, and intervened in the specific region with a highly targeted strategy.

The physical diagram of the whole system is shown in Figure 2b. The acquisition device contains sEMG acquisition module, air pressure monitoring module, and contact impedance monitoring module. The matched real-time signal processing interface provides the Glazer protocol voice prompt, real-time selective sEMG display, air pressure value display, poor interface contact warning, and abdominal/gluteus maximus muscle participation. After the test, the evaluation results are displayed on the interface. More details of data acquisition device and the real-time evaluation interface can be found in Note S2 and Figure S2 to Figure S3.

The parameters are related to specific contractions in the Glazer protocol [29]. The Glazer protocol consists of five steps: (a) A resting state for 1 min; (b) five rapid contractions with an interval of 10 s between each contraction, which evaluates the functional characteristics of the type-II muscle; (c) five tonic contractions of 10 sec, which evaluates intermittent characteristics of type-I and type-II muscles. There is a 10 s interval between two contractions as well. (d) A single 1 min continuous endurance contraction is followed to evaluate the functional characteristics of type-I muscle; (e) a resting state for 1 min.

As shown in Figure 2c, we use activation time (AT) and pressure increasing ratio (PIR) to evaluate PFM feedback to the voice command. AT is defined as the delay time between receiving the voice command and the muscle contraction [30]. PIR is defined as the growth rate of the air pressure in the contraction phase compared with the initial static air pressure. Contraction pressure value is reflected as the maximum value in rapid contraction, and as the average value in tonic and endurance contraction.

As shown in Figure 2d, regional functions are presented in a contour map. The parameter values are displayed in different colors in the map, and the abnormal region was easy to find. Several key parameters are proposed to comprehensively diagnose abnormal regions and functions of type-I muscle fiber (continuous contraction) and type-II muscle fiber (rapid contraction). Tonic contraction potential (TCP) is normally selected to evaluate the type-I muscle strength in clinical practice [31]. It is defined as the averaged amplitude of five 10-s contraction sEMG. Ratio of endurance contraction (REC) was selected to present the fatigue of type-I muscle [32]. It is defined as the averaged amplitude ratio of the last 10 s to the first 10 s contraction signals, in the 1 min endurance contraction phase. Rapid contraction potential (RCP) is commonly used for assessing type-II muscle strength [32], while variability of rapid contractions (VRC) was used for evaluating the stability of type-II muscle. The former is defined as the averaged amplitude of five maximum voluntary rapid contraction signals, while the latter is defined as the variance of five maximum voluntary rapid contractions.

As shown in Figure 2e, abnormal region was selected as the special region, then inter-region correlations between the special region and its nearby regions were evaluated. We divide the original sEMG signal into separated sections according to different muscle functions, and calculate the Pearson coefficient between the specific region and its nearby region in the selected sEMG waveforms, as the correlation value. When the correlation value is positive and close to 1, it indicates the two regions are in close positive cooperation; when the correlation value is close to −1, it means that regions are in negative cooperation.

### 2.4. Experimental Protocol

The detailed procedure of the clinical trial is as follows:

(1) The participant lies on her back with the hip and knee joints relaxed by 45°.

(2) The sterilized ASEA is placed into the participant’s vagina in a designated direction. Meanwhile, a standard electrocardiograph (ECG) electrode is also placed on the participant’s abdominal bone area (unilateral anterior superior iliac spine) as a reference electrode.

(3) Inflate the ASEA with the pressure monitored by the pressure sensing module. When the participant feels that the ASEA is tightly attached to muscles, the pressure value was set as the baseline for the subsequent process. The contact impedance of the electrode-muscle interface is then checked by the impedance module to ensure the electrical units and the probe work in the best conditions.

(4) Conduct the standard Glazer protocol flow. If abnormal muscle contraction was detected, the participant was given guidance for the proper way of exerting force.

(5) Deflate the ASEA and remove it out of the participant’s vagina. Properly handle the waste disposable ASEA.

(6) Evaluate mechanical properties of PFM, diagnoses functional abnormity of regional PFM, and calculate the correlation value of the specific region and its nearby regions.

(7) Confirm the diagnosis results, and propose targeted intervention strategy.

### 2.5. Statistical Analysis

Data are expressed as a mean ± standard deviation. We apply either the unpaired samples t-test or the Mann-Whitney test, depending on the statistical distribution of the data, to determine whether there are differences between the two columns of parameters. The star (*) indicates the level of statistical significance (*p*) < 0.05. Error bars represent 95% confidence interval. All data statistics are analyzed by Matlab (Version 2021b) and SPSS (Version 25.0).

## 3. Results

### 3.1. Accurate Signal Acquisition of ASEA

**Figure 3:**
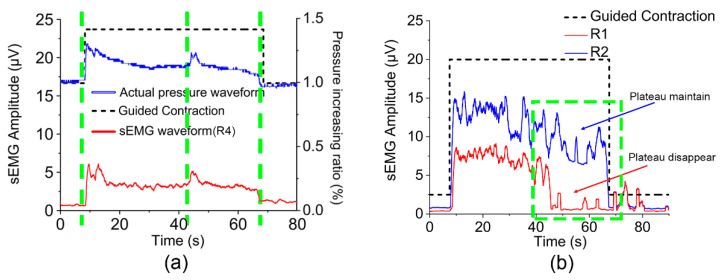
ASEA real-time waveform acquisition. The black dotted line represented the guided reference endurance contraction action. (**a**) Synchronous air pressure, MC-sEMG signal acquisition (by a participant in group A). The red line represented the sEMG signal collected in R4 region, and the blue line represented the air pressure signal. The green dotted line shows the time sequence consistency and details coincidence of sEMG signal and air pressure signal. (**b**) The sEMG waveform differentiation of endurance contraction of nearby muscle regions (by a participant in group B). The green frame showed the difference of muscle endurance status between the two adjacent regions, proving the detail of ASEA acquisition.

Figure 3a shows part of the data collected by ASEA during the endurance contraction. In the protocol process, the air pressure and MC-sEMG signals were collected by ASEA at the same time. The two types of signals have strong consistency on the time axis, and the details are almost the same, which shows the synchronization and accuracy of ASEA acquisition. Figure 3b shows the sEMG signal of the Endurance Contraction in the adjacent R1 and R2 regions. We found that from 40 s, the plateau of R2 region declined rapidly, while R1 region was maintained in the plateau, which showed that the participant’s continuous contraction function in R2 region was deteriorated, so it was meaningful to diagnose the abnormal function of regional PFM and implement targeted intervention strategy.

### 3.2. Pressure Feedback Analysis

As shown in Table 2, there were significant differences in PIR between the two groups, which indicated that the participants in group A exerted more powerful extrusion on ASEA, reflecting that their PFM strength are better than that of group B women. The AT between the two groups was also significantly different. The participants in Group A had faster response to voice commands, reflecting their better control of muscles. The results of pressure feedback preliminarily evaluated PFM function difference between the two groups, and also proved the rationality of grouping.

### 3.3. Type-I Muscle Function

**Figure 4:**
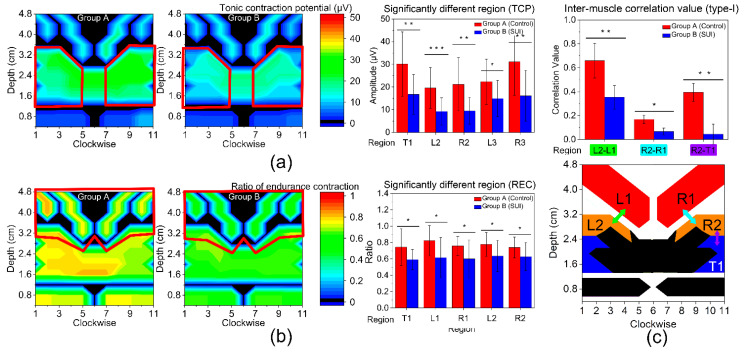
Type-I muscle function evaluation of two groups. (**a**) Tonic contraction potential (TCP) and (**b**) ratio of endurance contraction (REC) for each group. From left to right: amplitude results contour map of group A; amplitude results contour map of group B (red frame marks the significantly different regions); significantly different regions of parameter, including T1, L2, R2, L3, R3. According to the ultrasonic-assisted measurement, the muscle relationship corresponding to these regions are: T1: US; L2, R2: PC; L3, R3: PR. Asterisk indicates significant difference. *: *p* < 0.05; **: *p* < 0.01; ***: *p* < 0.001. (**c**) Inter-region correlation value of type-I muscle fiber. The upper graph is the significantly different region of correlation value and the lower is the location diagram.

Table 3 and Figure 4a are the TCP results for the two groups. TCP was found to be significantly different in T1, L2, R2, L3, and R3 regions, indicating that the volunteers in group A have better type-I muscle strength in these regions. As shown in Figure 4b, REC was found to be significantly different in T1, L1, R1, L2, and R2 regions between the two groups, indicating that fatigue happens easily in these regions for the volunteers in group B.

Based on the above results, L2 and R2 regions were selected as the specific regions for the following assessment. Figure 4c shows the inter-region correlation between the specific regions and their nearby regions. It was found that the correlation value between L2 and L1 (0.660 ± 0.146 vs. 0.354 ± 0.099, *p* = 0.007) is significantly different, and the correlation value of the women in group A is higher compared to those of group B, implying that women in group A have better muscle synergy in these regions. The relationship between R2 and the nearby regions was also calculated. The results show that the group A women have a closer muscle correlation in R2-R1 (0.168 ± 0.034 *vs.* 0.068 ± 0.028, *p* = 0.033) and in T1 (0.395 ± 0.075 vs. 0.045 ± 0.084, *p* = 0.004).

### 3.4. Type-II Muscle Function

**Figure 5:**
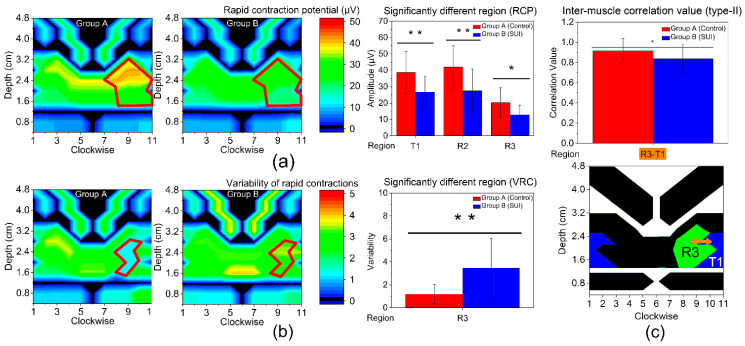
Type-II muscle function evaluation of two groups. (**a**) Rapid contraction potential (RCP) and (**b**) variability of rapid contractions (VRC) for each diagnosis group. From left to right: amplitude results contour map of group A; amplitude results contour map of group B (red frame marks the significantly different regions); significantly different regions of parameter, including T1, R2, R3. According to the ultrasonic-assisted measurement, the muscle relationship corresponding to these regions are: T1: US; R2: PC; R3: PR. Asterisk indicates significant difference. *: *p* < 0.05; **: *p* < 0.01. (**c**) Inter-region correlation value of type-II muscle fiber. The upper graph is the significantly different region of correlation value and the lower is the location diagram.

Table 4 and Figure 5 show the results of type-II muscle function. The RCP contour maps of two groups are shown in Figure 5a. The results show that RCP is found to be significantly different between the two groups in T1, R2, R3 regions, indicating that the women in group A have a stronger explosive power in these regions. As shown in Figure 5b, VRC is also found to be significantly different in R3 region, suggesting that the women in group B have unstable muscle coordination.

Then R3 region was selected as the specific region, and the inter-region correlation of rapid contraction phase between R3 and its nearby region were calculated, as shown in Figure 5c. It was found that correlation value between R3 and T1 is much higher in group A compared to that of group B (0.915 ± 0.122 vs. 0.837 ± 0.145, *p* = 0.037), implying women in group A have better muscle synergy in these regions. The results of type-II inter-region correlation present a considerable asymmetry.

## 4. Discussion

This study is the first attempt to objectively, comprehensively, and quantitatively diagnose the regional PFM functional abnormalities between the postpartum asymptomatic women and the PSUI women, with the ASEA-based multifunctional evaluation technology. The ASEA successfully collected the air pressure signal and the MC-sEMG signals, and the system perfectly implemented the evaluation of the PFM multifunctional characteristics and diagnosis of malfunctions of regional PFM, which cannot be achieved by using the existing technologies. Results showed that there were significant differences between the two groups in pressure feedback, regional PFM function, and inter-region correlation. In addition, no women reported pain or any discomfort during the whole process, and they believed that the use of ASEA was more conducive to PFM complete contraction than the existing probe, which showed the superiority, safety, and repeatability of our technology.

The MET accurately processed muscle feedback to the airbag and evaluated PFM function. Our study found that women in group A had faster muscle response than that of the women in group B, and women in group A had higher pressure increasing ratio during contraction. All relevant parameters between the two groups had statistical differences. Relevant research pointed out that as an important part of the pelvic floor support functions, the decrease in strength is closely related to PSUI development [33,34].

The MET innovatively acquired the detailed information of two type muscle fiber functions according to the sEMG-based parameters. For the type-I muscle function evaluation of regional PFM, the statistical results show that women in group B had weaker type-I strength in T1, L2, L3, R2, and R3 regions, were prone to fatigue in T1, L1, L2, R1 and R2 regions, and had worse muscle synergy in L1-L2, R1-R2, R2-T1, compared to group A. In terms of physiological anatomy, IC and PC are the main components of levator ani muscle. Both IC and PC are the important muscles supporting pelvic organs such as urethra and bladder [35,36]. When the type-I muscle functional abnormality occurs in IC and PC, they will not be able to undertake enough support, resulting in changes of the anatomical position of the bladder and urethra, and then leading to abnormal urination function. Therefore, the abnormal type-I muscle function at IC and PC in group B is related to SUI symptoms. For the type-II muscle function evaluation of regional PFM, we found that women in group B had weaker type-II strength in T1, R2, R3 regions, had more unstable muscle manipulation in R3 regions, and had worse muscle synergy in R3-T1, compared to group A. According to the ultrasonic-assisted measurement, the muscle relationship corresponding to these regions are: R2: PC; R3: PR; T1: US. In terms of physiological anatomy, the PR is a part of the PC, which is formed by the muscle fibers of the PC that wrap the rectum from the beginning. According to the basic theory of SUI-hammock theory, the closure of the urethra is caused by the contraction of the first half of PC to form the so-called hammock, and increased bladder neck activity leads to SUI [37,38]. US functional dysfunction is one of the pathophysiological mechanisms of SUI. In SUI patients, PR muscle defect was the strongest contribution to the PFM loss and inverse [39]. It is consistent with the PR anomaly we have found in B group. In addition, urination control depends on the restraining effect of the US [40,41,42]. In our study, the synergetic relationship between type-I and type-II muscles of US and surrounding muscle was decreased, which may be the cause of PSUI.

The MET established the evaluation of the dynamic inter-region correlation of PFM. We successfully separated all participants’ sEMG signals according to different steps dominated by type-I and type-II muscle fiber, and calculated the Pearson coefficient between different regions. We found that women in group A have a larger absolute Pearson coefficient value than women in group B, and the regions with significant differences in correlation values have strong consistency with those in sEMG parameters. Relevant research showed that MRI examination also detected asymmetry of levator ani muscle morphology in SUI patients [11]. The consistency of evaluation results shows that the Pearson coefficient calculated from sEMG waveforms can be one of the evaluation parameters to characterize muscle function. The occurrence of PSUI may be related to the bad muscle synergy, the training of improving the coordination between muscle regions may be a useful strategy to help recover and prevent PSUI. To our knowledge, there is no report yet on malfunctions of regional PFM and correlation of specific regions.

These results proved that the ASEA-based evaluation technology can not only reliably evaluate the multifunctional PFM function, but also further obtain the details of malfunctions of regional PFM, which can provide valuable reference information and advanced treatment concepts for clinical practices, such as assessing pelvic muscle damages for delivery related dysfunction or differential treatment for specific regions.

## 5. Conclusions

In summary, MET is a novel clinical evaluation technology for real-time diagnosis of malfunctions of regional PFM. The ASEA synchronously collects high-quality air pressure and MC-sEMG signals, then the matched system feedbacks the details of pressure response, innovatively assessed functional abnormalities of type-I and type-II muscles, and the correlation between muscle groups in real time. The consistency between the clinical experimental results and the SUI pathogenesis theory proved the great reliability of diagnosing PSUI-related muscle abnormality with MET. In addition, we successfully found that the abnormal functions of PSUI women are concentrated in specific muscle regions, which have weak correlation with the nearby muscle groups. These details with clinical significance are vitally important for targeted treatment strategy and cannot be accessed by existing technologies. Compared with the previously reported work, the MET provides a convenient and fast way to diagnose the abnormal function of regional PFM in detail. It is our expectation that the MET could tightly link the clinical point-of-care diagnostics and pathological research, assist in the formulation of the real-time point-to-point treatment after diagnosis, and explore the pathology in detail, so as to promote the development of PFD diagnostics research.

## Figures and Tables

**Table 1:** Baseline demographics of participants, postpartum and asymptomatic, control (Group A), PSUI participants (Group B).

Variables	Group A N = 15	Group B N = 15	*p*-Value
Age (years)	29.80 ± 4.63	29.33 ± 4.94	0.819
BMI (kg/m^2^)	21.82 ± 2.18	22.81 ± 2.94	0.174
Waist-hip ratio	0.90 ± 0.05	0.88 ± 0.05	0.503
No. of births (*n*)	1.40 ± 0.51	1.33 ± 0.49	0.478

**Table 2:** Comparison of pressure-related parameters, control (Group A), PSUI participants (Group B).

Variables	Group A N = 15	Group B N = 15	*p*-Value
Rapid Contra. PIR (%)	2.66 ± 1.85	1.34 ± 0.91	0.023
Tonic Contra. PIR (%)	1.14 ± 0.58	0.41 ± 0.22	<0.001
Endurance Contra. PIR (%)	1.13 ± 0.68	0.57 ± 0.40	0.012
Rapid Contra. AT (s)	0.80 ± 0.28	1.16 ± 0.36	0.006
Tonic Contra. AT (s)	0.70 ± 0.28	1.12 ± 0.38	0.002
Endurance Contra. AT (s)	0.90 ± 0.36	1.20 ± 0.38	0.036

**Table 3:** Type-I muscle function assessment results of participants, postpartum and asymptomatic, control (Group A), PSUI participants (Group B).

	Tonic Contraction Potential (TCP), μV			Ratio of Endurance Contraction (REC)		
Region	Group A N = 15	Group B N = 15	*p*-Value	Group A N = 15	Group B N = 15	*p*-Value
T1 (US)	30.15 ± 14.19	16.78 ± 8.73	0.004	0.74 ± 0.22	0.59 ± 0.12	0.028
B1 (EAS)	17.33 ± 9.26	14.47 ± 8.86	0.396	0.71 ± 0.12	0.61 ± 0.16	0.063
L1 (IC)	12.95 ± 7.10	12.12 ± 10.30	0.800	0.82 ± 0.19	0.62 ± 0.25	0.014
R1 (IC)	12.18 ± 4.24	10.27 ± 8.42	0.438	0.76 ± 0.12	0.60 ± 0.23	0.025
L2 (PC)	19.57 ± 8.94	9.20 ± 6.06	<0.001	0.78 ± 0.15	0.64 ± 0.19	0.029
R2 (PC)	21.17 ± 11.72	9.48 ± 5.89	0.002	0.74 ± 0.13	0.63 ± 0.17	0.049
L3 (PR)	22.36 ± 9.96	14.90 ± 7.98	0.031	0.78 ± 0.14	0.70 ± 0.33	0.363
R3 (PR)	31.14 ± 14.75	16.12 ± 11.22	0.004	0.67 ± 0.12	0.64 ± 0.15	0.583
L4 (VS)	4.12 ± 1.79	3.67 ± 1.44	0.452	0.75 ± 0.28	0.65 ± 0.21	0.274
R4 (VS)	4.03 ± 1.85	3.73 ± 1.36	0.619	0.74 ± 0.34	0.64 ± 0.29	0.434

**Table 4:** Type-II muscle function assessment results of participants, postpartum and asymptomatic, control (Group A), PSUI participants (Group B).

	Rapid Contraction Potential (RCP), μV			Variability of Rapid Contractions (VRC)		
Region	Group A N = 15	Group B N = 15	*p*-Value	Group A N = 15	Group B N = 15	*p*-Value
T1 (US)	38.66 ± 12.75	26.75 ± 9.66	0.008	2.01 ± 1.08	3.08 ± 2.55	0.145
B1 (EAS)	27.02 ± 10.87	22.97 ± 9.28	0.282	3.38 ± 2.64	4.04 ± 2.92	0.526
L1 (IC)	13.76 ± 11.20	11.96 ± 6.06	0.588	1.45 ± 1.35	1.79 ± 1.31	0.485
R1 (IC)	10.57 ± 3.40	9.79 ± 5.08	0.628	1.26 ± 0.96	1.53 ± 1.11	0.480
L2 (PC)	21.15 ± 10.06	20.72 ± 12.59	0.918	1.91 ± 1.63	1.81 ± 1.79	0.881
R2 (PC)	41.96 ± 13.08	27.50 ± 13.38	0.006	1.70 ± 1.61	1.66 ± 1.46	0.066
L3 (PR)	33.77 ± 16.50	25.52 ± 10.04	0.109	2.34 ± 1.54	2.96 ± 2.73	0.446
R3 (PR)	20.23 ± 9.13	12.82 ± 5.95	0.014	1.19 ± 0.83	3.46 ± 2.57	0.003
L4 (VS)	9.24 ± 4.05	9.20 ± 3.58	0.975	1.07 ± 0.64	1.27 ± 0.59	0.375
R4 (VS)	8.87 ± 3.54	8.36 ± 3.16	0.682	1.48 ± 1.29	1.35 ± 0.82	0.732

## Data Availability

Data are available upon a request to the authors.

## Supplementary Materials

The following supporting information can be downloaded at: https://www.mdpi.com/article/10.3390/diagnostics13061158/s1. Figure S1: The physical picture of ASEA probe and existing intravaginal probes. Figure S2: Real-time signal processing interface during Glazer protocol. Figure S3: Real-time evaluation feedback interface after Glazer protocol. Table S1: The comparison between ASEA probe and existing intravaginal probes. Table S2: The cost comparison between ASEA probe and existing devices (per test). Note S1: Existing intravaginal probes for recording sEMG signal. Note S2: The data acquisition device and real-time signal processing interface.
