# Supplementary material for: Multifunctional Evaluation Technology for Diagnosing Malfunctions of Regional Pelvic Floor Muscles Based on Stretchable Electrode Array Probe
Source: Diagnostics (Basel). 2023 Mar 17;13(6):1158. doi: 10.3390/diagnostics13061158 (PMC10047914; doi:10.3390/diagnostics13061158)
Supplement: Supplementary file 1 [file diagnostics-13-01158-s001.zip › diagnostics-2269255-supplementary.pdf]

diagnostics

Supplementary Material

Note S1. Existing intravaginal probes for recording sEMG signal.

Existing intravaginal probes used in clinical practice and research can be divided into two types: rigid dual-channel probes and high-density array probes.

The rigid probe with a dual-channel electrode has been developed to evaluate female PFM in clinical, which has the merits of non-invasive way and convenient operation [1-3]. However, its fixed shape and dimensions have poor deformability, and its contact pads are unable to make good contact with some targeting muscles for proper assessment due to the irregular shape of the vagina. More importantly, PFD is mostly related to several muscles' dysfunction, therefore a large number of electrodes and contact pads or multi-parameter measurements are necessary for precise and accurate identification of damaged muscles. The small number of contact pads makes the dual-channel sEMG assessment impossible to meet the requirements we mentioned above. Examples of dual-channel rigid probes can be found in Figure S1a.

Recently, a high-density electrode array-based sEMG (HD-sEMG) assessment method has been proposed to assess PFM status by using a large number of contact pads on the surface of the probe, providing an innovative concept for a more accurate diagnosis of pelvic floor electrophysiology. Cescon et al. evaluated the innervation effect of delivery-related trauma by a means of intra-anal sEMG performed with sixteen silver contact pads equally spaced along the circumference on the surface of rigid probe device [4]. Peng Yun et al. examined the feasibility of utilizing a high-density intra-vaginal sEMG recording approach to characterize pelvic muscle activities [5]. Dias et al. assessed the feasibility of objectively assessing pelvic floor hypertonicity in women with interstitial cystitis/bladder pain syndrome (IC/BPS) using an intra-vaginal high-density sEMG probe [6]. The contact pads of high-density rigid probe are arranged in array mode, thus remain mixed signals from different muscles, resulting in poor accuracy of the collected sEMG signal. For accurate and precise measurement of muscle potentials using sEMG, a large number of bipolar electrode pairs placed along the multiple fiber directions would be the best scenario. As same as the rigid dual-channel probe, the rigid material of high-density probe makes the contact pad unable to deform and fit tightly with the muscle, which may lead the interference signals into sEMG signals. Examples of high-density rigid probes can be found in Figure S1b [7].

The details of existing intravaginal probes shown in Table S1 [2; 4; 5].

Figure S1. The physical picture of ASEA probe and existing intravaginal probes. (a) Rigid dual-electrode probe. Left: Femelex probe; Right: InCare Vaginal Probe 9597. (b) High-density array probe. Left: Disposable rectal probe; Right: TMSi High-Density probe. (c) The ASEA probe.

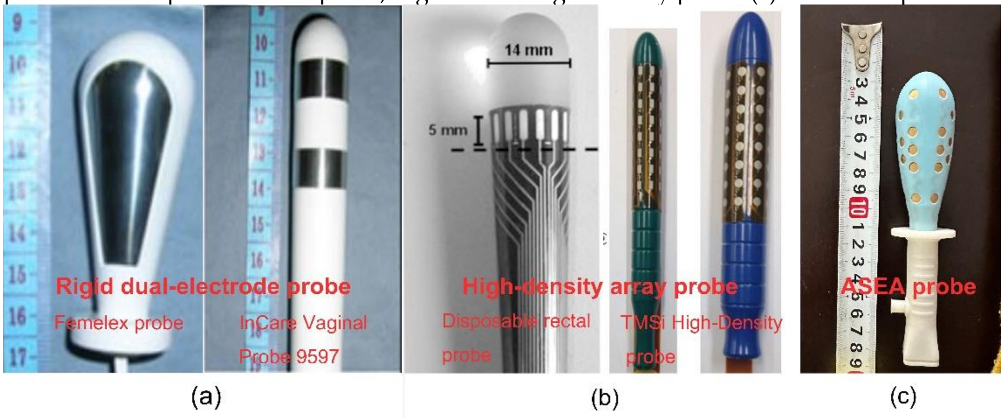

Table S1. The comparison between ASEA probe and existing intravaginal probes.

Probe	Shape	Material hardness	Contact pad size	Contact pad location	Contact pad configuration
Femelex probe	Pear-shaped	rigid	2.5 cm in length	2 contact pads located at the opposite side	bipolar
InCare Vaginal Probe 9597	Cylindrical	rigid	0.9 cm wide	2 circumferen-tial stainless steel contact pads	monopolar
Disposable rec-tal probe	Cylindrical	rigid	0.5 cm length	16 contact pads equally spaced along the cir-cumference	monopolar
TMSi High-Density probe	Cylindrical	rigid	0.4 cm diame-ter	64 array con-tact pads	monopolar
ASEA probe	Pear shaped airbag	35.6% deforma-ble	0.5 cm diame-ter	32 contact pads placed along the multiple fi-ber directions	bipolar

Table S2. The cost comparison between ASEA probe and existing devices (per test).

**Technology**	**System Size**	**Money Consumption (RMB)**	**Evaluation Duration**	**Human Resources**
MET	suitcase size	40-60	20mins	Trained staff
Dual-channel sEMG probe	Refrigerator size	70-110	30mins	Trained staff
PFM Ultrasound	Refrigerator size	200-300	15mins	Two skilled doctors
Magnetic resonance imaging	Bed size	600-800	10mins	Skilled doctor

Note S2. The data acquisition device and real-time signal processing interface**.**

The data acquisition device contains a sEMG acquisition module, an air pressure monitoring module, and an impedance monitoring module. The sEMG acquisition module uses ADS1299 (Texas Instruments, The United States) as the core microchip. The module is able to maintain stable sEMG signal acquisition and transmission under 32-channel simultaneous sampling (1K sampling rate). XGZP6847A (CFSensor, China) is selected as the air pressure sensing module which can measure the internal air pressure of ASEA with 1.5% accuracy. The impedance monitoring module checks the contact impedance between the contacting pad and muscle, to ensure close contact between all the electrical units and the corresponding muscles.

The matched real-time signal processing interface provides the Glazer protocol voice prompt and corresponding contraction animation so that subjects can better understand the action. During the test, it can selectively watch the sEMG signals of any PFM region, and is able to feed back the contact condition of the probe-muscle interface in real time. At the same time, it can detect the contraction participation of the abdominal muscle and gluteus maximus muscle. Real-time air pressure is also displayed on the interface, as shown in Figure S2. After the test, the regional PFM evaluation results will be displayed on the interface for doctors to analyze, as shown in Figure S3.

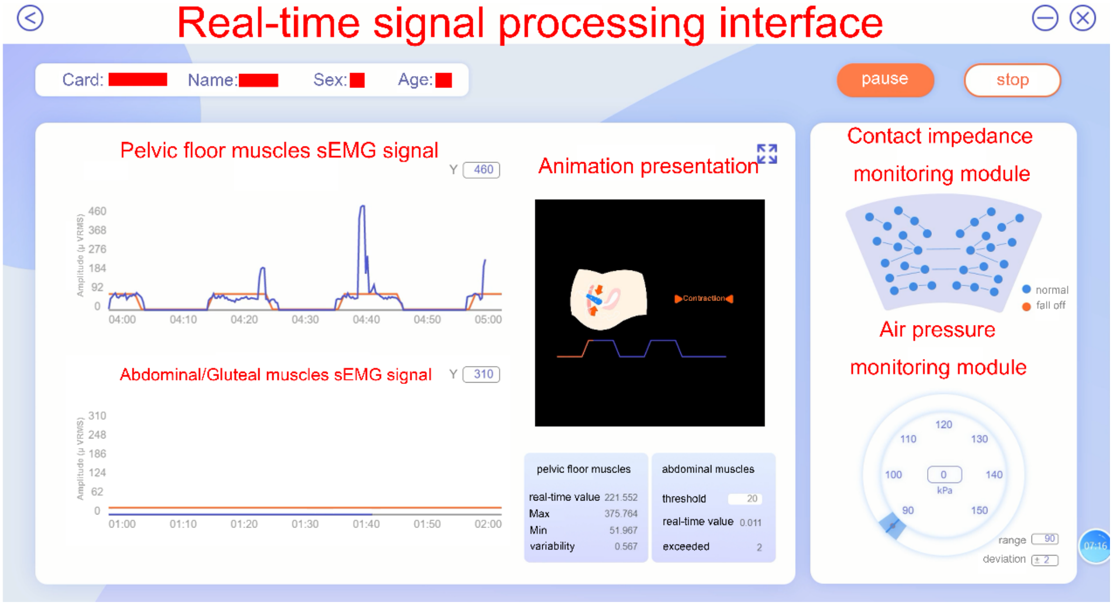

Figure S2. Real-time signal processing interface during Glazer protocol.

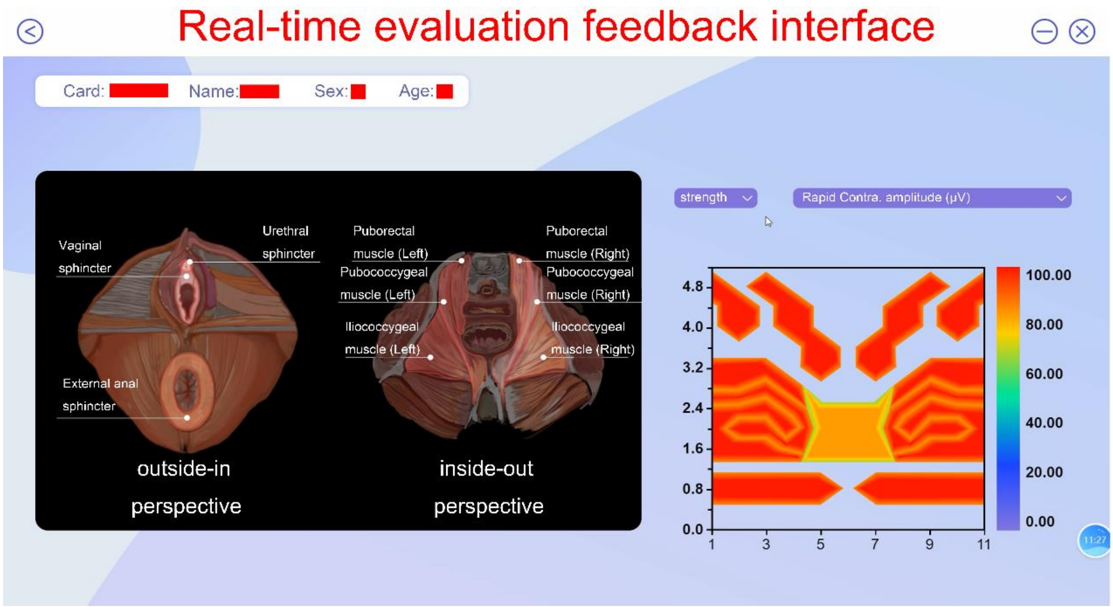

Figure S3. Real-time evaluation feedback interface after Glazer protocol.
